# Comment on: “Pecto-intercostal Fascial Block for perioperative pain management in patients undergoing open cardiac surgery”

**DOI:** 10.1186/s12871-022-01794-3

**Published:** 2022-08-19

**Authors:** Raghuraman M. Sethuraman

**Affiliations:** Department of Anesthesiology, Sree Balaji Medical College and Hospital, BIHER, #7, Works Road, New colony, Chromepet, 600044 Chennai, India

**Keywords:** Pecto-intercostal fascial block, Median sternotomy, Pain relief

## Abstract

This article (Correspondence) is in response to the recently published article on the role of Pecto-intercostal Fascial Block for cardiac procedures by Zhang et al. in “BMC Anesthesiology”. I greatly appreciate the authors for publishing this study in which Pecto-intercostal Fascial Block, a novel technique for providing pain relief in open cardiac surgical procedures was evaluated. I wish to present my reflections on this article as well as to add a few more points on this topic.

Dear Editor,

I read the recently published article that has analyzed the effectiveness of Pecto-intercostal Fascial Block (PIFB) for patients undergoing open cardiac surgery [1] with keen interest. I greatly appreciate Zhang et al. for publishing this study in which PIFB, a novel technique for providing pain relief in open cardiac surgical procedures was evaluated [1]. I wish to present my comments on this article as well as to add a few more points on this topic.

Zhang et al. [1] have stated in the “Discussion” section that their study was the first double-blind, randomized, controlled trial on PIFB in open cardiac surgeries. However, to my knowledge, two studies were published on the evaluation of PIFB in open cardiac procedures within a short span in July 2020 [2, 3]. Khera et al. have investigated the role of PIFB in patients undergoing various open cardiac procedures through a median sternotomy and observed that PIFB reduced the cumulative opioid consumption although it was not statistically significant [2]. They provided the block within 2 h of admission to the intensive care unit (ICU) postoperatively, as well as on the first postoperative day [2]. Another study by Kumar et al. has also applied PIFB in the same population and observed a significant reduction in opioid requirement when compared to the control group [3]. This was a single-blind study in which the postoperative nurses who monitored the outcomes were unaware of whether the block was performed or not, while the anesthesiologists performed the PIFB without any placebo group before transferring the patients to ICU [3]. In contrast to these studies [2, 3], Zhang et al. have administered the PIFB before the surgery [1].

Zhang et al. [1] have also stated that the concentration and volume of local anesthetic (20 ml of ropivacaine 0.4% on each side) used in this study were based on previous research without citing any reference.

The control group of patients had significantly higher levels of blood glucose despite a significant rise in insulin levels, which was attributed as “Insulin resistance”, by Zhang et al. [1]. Also, they mentioned that insulin resistance resulted in a significant difference in the IL-6 (interleukin-6) levels. However, it is not clear whether the increased levels of IL-6 can be attributed to insulin resistance that too on an acute basis [4]. Therefore, I believe that elevation of blood glucose levels in the control group could be probably due to the rise of stress hormones particularly steroids, which were unfortunately not estimated in this study.

Zhang et al. [1] have mentioned that including only the valve replacement procedures in this study was one of the limitations. However, it can be considered as “strength” also as the purpose of evaluating PIFB is mainly for pain related to median sternotomy and it would not cover the pain associated with chest drains, graft sites that happen in other cardiac surgeries although these are not as severe as sternotomy pain. Rather, this study is unique in that aspect as well as for estimation of IL-6, insulin resistance unlike the previously published studies [2, 3] and congratulate Zhang et al. [1] for the same.

## Data Availability

Not Applicable.

## Abbreviation

PIFB: Pecto-intercostal Fascial Block
